# Identifying drug-pathway association pairs based on L_2,1_-integrative penalized matrix decomposition

**DOI:** 10.1186/s12918-017-0480-7

**Published:** 2017-12-14

**Authors:** Jin-Xing Liu, Dong-Qin Wang, Chun-Hou Zheng, Ying-Lian Gao, Sha-Sha Wu, Jun-Liang Shang

**Affiliations:** 1 School of Information Science and Engineering, Qufu Normal University, Rizhao, China; 2 Library of Qufu Normal University, Qufu Normal University, Rizhao, China

**Keywords:** Drug discovery, Sparse method, Integrative penalized matrix decomposition, L_2,1_-norm penalty

## Abstract

**Background:**

Traditional drug identification methods follow the “one drug-one target” thought. But those methods ignore the natural characters of human diseases. To overcome this limitation, many identification methods of drug-pathway association pairs have been developed, such as the integrative penalized matrix decomposition (iPaD) method. The iPaD method imposes the L_1_-norm penalty on the regularization term. However, lasso-type penalties have an obvious disadvantage, that is, the sparsity produced by them is too dispersive.

**Results:**

Therefore, to improve the performance of the iPaD method, we propose a novel method named L_2,1_-iPaD to identify paired drug-pathway associations. In the L_2,1_-iPaD model, we use the L_2,1_-norm penalty to replace the L_1_-norm penalty since the L_2,1_-norm penalty can produce row sparsity.

**Conclusions:**

By applying the L_2,1_-iPaD method to the CCLE and NCI-60 datasets, we demonstrate that the performance of L_2,1_-iPaD method is superior to existing methods. And the proposed method can achieve better enrichment in terms of discovering validated drug-pathway association pairs than the iPaD method by performing permutation test. The results on the two real datasets prove that our method is effective.

## Background

Studies of the mechanism of carcinogenesis have led to the implementation that cancer is radically a disease of a variety of genetic aberrations [1]. And at present, the main method to treat cancer is drug therapy. New drug research is an important topic of the drug discovery. And one of the basic research concept of these new drugs is to determine the interaction between drugs and targets. And it can be used to predict candidate drugs, which may act on targets [2]. Besides under the guidance of the concept of pharmacology research and development for new drugs, it can also be used for the relocation of existing drugs, and to forecast the new targets for known drugs [3]. Drug discovery technology is in primary stage, but many related algorithms have been developed to find drug targets. In general, original drug target identification algorithms follow the “one drug-one target” line [4]. The purpose of those methods is to discover the effective drugs, which act on individual targets. It is obvious that those methods do not take into consideration of the relations among genes. Thus, “one drug-one target” algorithms ignore related pathways [5]. Generally, many complex diseases are resulted from unique pathway functions rather than individual genes. And the function of drugs is not just aiming at single proteins, but rather affecting the complex interaction of some associated biological pathways [6]. Therefore, identifying drug-pathway associations is a momentous task for quickening the development of drug discovery.

With the rapid development of high-throughput drugs and pathways related data, it is feasible for researchers to infer drug-pathway interactions. A large amount of studies have utilized the drug related data to obtain insights on drug-pathway modes of action [7]. Gene Set Enrichment Analysis (GSEA) is a traditional method to identify drug-pathway associations. GSEA is proposed by Harvard University and MIT’s broad institute research group. It is utilized to analyze genome-wide expression microarray and drug related data. You can download it after free register [8], whose website is http://software.broadinstitute.org/gsea/index.jsp. Based on known gene-pathway association information, the GSEA method can forecast responsiveness of pathways. But the GSEA method does not consider the known pathway information, its identification precision is poor [9]. In order to improve the identification precision and use the prior information, the FacPad method is proposed to predict drug-pathway associations, and it build a sparse Bayesian factor analysis model to infer pathway responsive for drug treatments [6]. In order to further improve the performance of the FacPad method, another Bayesian model named “iFad” is developed to discover the novel drug-pathway associations [10]. And Ma et al. apply the iFad method to analyze gene expression and drug related data from the NCI-60 cell lines. The NCI-60 cell lines is from the NCI-60 project, which provides useful information for various types of “Omics” characterization of 60 human cancer cell lines with nine different cancer types. The iFad method can discover effective drug-pathway associations. However, its computational costing is expensive since this method applies the Markov Chain Monte Carlo (MCMC) algorithm [11] to perform statistical inferences. At the same time, some prior parameters in the iFad model require to be specified in advance by the investigators. With the rapid development of modern genomics and pharmacology technologies, the dimensionality of the raw data becomes larger and larger, that is, these data have a large number of variables [12]. Thus, the size of sample is also becoming larger and larger. And the computational expense of dealing with the high-dimensional data becomes more expensive. Based on the above problems, an efficient method named “iPaD” is proposed to analyze drug related data [13]. Li et al. use integrative penalized matrix decomposition (iPaD) method to jointly analyze drug expression and drug sensitivity data. And Li et al. apply the iPaD method to the Cancer Cell Line Encyclopedia (CCLE) and NCI-60 datasets. Compared with the NCI-60 data set, the CCLE data set has the larger sample size. At the moment, the CCLE project has more than 1000 cell lines. Compared with the iFad method, the iPaD method has obvious superiority in computational efficiency. And the iPaD method only has one parameter required to be turned. In addition, the iPaD method applies the *L*_1_-norm penalty to obtain sparse solutions. However, the sparsity produced by*L*_1_-norm penalty is too dispersive [14].

In this paper, we impose the *L*_2, 1_-norm penalty to replace the *L*_1_-norm penalty on the drug-pathway association matrix. The *L*_2, 1_-norm regularization penalty can make each row of the drug-pathway association matrix as a whole and produce row sparsity solutions [15, 16]. Besides, the *L*_2, 1_-norm penalty can select the most prominent morphometric variables [17]. In this paper, compared with the iPaD method, our new proposed method has two outstanding advantages: firstly, the *L*_2, 1_-iPaD method can achieve better performance in identifying validated drug-pathway associations by applying our proposed method to the CCLE and NCI-60 datasets; secondly, in this paper, we also perform permutation test to evaluate the significance of the identified drug-pathway associations, the experimental results demonstrate that our proposed method can gain the smaller *P*-values. Thus, we can obtain that our proposed method can achieve better overall enrichment in terms of identifying drug-pathway association pairs.

In the next subsection, at first, we will describe a novel algorithm named *L*_2, 1_-iPaD to identify drug-pathway associations. And then we will apply the *L*_2, 1_-iPaD method on two real datasets (the CCLE and NCI-60 datasets) and give the results of our proposed and iPaD methods. Finally, we will give the conclusions and future work.

## Method

### Model description

Given a gene expression data matrix **Y** ^(1)^ with the size of *N* × *G* ^(1)^ and a drug sensitivity data matrix **Y** ^(2)^ with the size of *N* × *G* ^(2)^. *N* denotes the number of samples, *G* ^(1)^ and *G* ^(2)^ denote the number of genes and drugs, respectively. The traditional iPaD method decomposes the gene expression matrix **Y** ^(1)^ into the pathway activity level matrix **X** ∈ *R* ^*N* × *K*^ and the gene-pathway interaction matrix **B** ^(1)^. *K* denotes the number of pathways. And the iPaD method decomposes the drug related data matrix **Y** ^(2)^ into the pathway activity level matrix **X** and the drug-pathway interaction matrix **B** ^(2)^. The model of iPaD method can be introduced as follows:

1 $$ {\displaystyle \begin{array}{l}{\mathbf{Y}}^{(1)}={\mathbf{XB}}^{(1)}+{\mathbf{E}}^{(1)}\\ {}{\mathbf{Y}}^{(2)}={\mathbf{XB}}^{(2)}+{\mathbf{E}}^{(2)},\end{array}} $$

where **E** ^(1)^ and **E** ^(2)^ denote the error matrices in (1). Then the model (1) can be written as the following form:

2 $$ \underset{\mathbf{X},{\mathbf{B}}^{(1)},{\mathbf{B}}^{(2)}}{\min }{\left\Vert {\mathbf{Y}}^{(1)}-{\mathbf{X}\mathbf{B}}^{(1)}\right\Vert}_F^2+{\left\Vert {\mathbf{Y}}^{(2)}-{\mathbf{X}\mathbf{B}}^{(2)}\right\Vert}_F^2. $$

In general, a drug is associated with a few pathways, therefore, drug-pathway association matrix **B** ^(2)^ is sparse. Based on this fact, in this paper, we propose a novel method to improve the performance of the iPaD method. We employ the *L*_2, 1_-norm regularization to replace the *L*_1_-norm regularization in the iPaD method. Then the optimization model of *L*_2, 1_-iPaD method can be written as follows:

3 $$ {\displaystyle \begin{array}{l}\underset{\mathbf{X},{\mathbf{B}}^{(1)},{\mathbf{B}}^{(2)}}{\min }{\left\Vert {\mathbf{Y}}^{(1)}-{\mathbf{X}\mathbf{B}}^{(1)}\right\Vert}_F^2+{\left\Vert {\mathbf{Y}}^{(2)}-{\mathbf{X}\mathbf{B}}^{(2)}\right\Vert}_F^2+\lambda {\left\Vert {\mathbf{B}}^{(2)}\right\Vert}_{2,1}\\ {}\mathrm{subject}\  \mathrm{to}\ \sum \limits_i{\mathbf{X}}_{i,j}^2\le 1,\forall j=1,\dots, K;\\ {}{\mathbf{B}}_{i,j}^{(1)}=0,\forall \left(i,j\right):{\mathbf{L}}_{i,j}^{(1)}=0,\end{array}} $$

where ‖**W**‖_*F*_ denotes the Frobenius norm of the matrix **W**. The detailed definition of the Frobenius norm can be written as ${\left\Vert \mathbf{W}\right\Vert}_F=\sqrt{\sum_{i=1}^m{\sum}_{j=1}^d{\mathbf{w}}_{i,j}^2}=\sqrt{\sum_{i=1}^m{\left\Vert {\mathbf{w}}^i\right\Vert}_2^2}$, where **w** ^*i*^ is the *i*-th row of the matrix **W**. ‖**W**‖_2, 1_ denotes the*L*_2, 1_-norm of the matrix **W**. The definition of *L*_2, 1_-norm is first proposed in reference [18]. And the *L*_2, 1_-norm has been applied in many research direction such as the feature identification [19, 20] and image direction [21, 22]. The definition of *L*_2, 1_-norm can be written as ${\left\Vert \mathbf{W}\right\Vert}_{2,1}={\sum}_{i=1}^m\sqrt{\sum_{j=1}^d{\mathbf{w}}_{i,j}^2}={\sum}_{i=1}^m{\left\Vert {\mathbf{w}}^i\right\Vert}_2$. Specifically, we firstly need to calculate the *L*_2_-norm of the vector **w** ^*i*^, and then compute the *L*_1_-norm of the vector *b*(**w**) = (‖**w** ^1^‖_2_, ‖**w** ^2^‖_2_, ⋯, ‖**w** ^*m*^‖_2_)^*T*^ [23]. The *L*_2, 1_-norm penalty achieves the rows sparsity of the drug-pathway association matrix **B** ^(2)^. Thus, the irrespective drug-pathway pairs can be abandoned. In addition, ${\mathbf{L}}_{i,j}^{(1)}\in \left\{0,1\right\}$ is a known prior knowledge matrix with the size of *K* × *G* ^(1)^. In the model of *L*_2, 1_-iPaD method, ${\mathbf{L}}_{i,j}^{(1)}$ is used for indicating gene-pathway association matrix **B** ^(1)^. When ${L}_{i,j}^{(1)}=1$, the *i*-th pathway will be associated with the *j*-th gene. When ${L}_{i,j}^{(1)}=0$, the *i*-th pathway will not be associated with the *j*-th gene. Thus, similar to reference [13], in order to merge the known pathway-gene relationship, we impose the first constraint on gene-pathway association matrix **B** ^(1)^. Besides, the second constraint on pathway activity level matrix **X** is used to guarantee that the optimization problem (3) is identifiable.

### Optimization algorithm

In this paper, the optimization model (3) is convex, that is, when **X** is fixed, optimizing gene-pathway association matrix **B** ^(1)^ and drug-pathway association matrix **B** ^(2)^ are both convex optimization problems. And when gene-pathway association matrix **B** ^(1)^ and drug-pathway association matrix **B** ^(2)^ are fixed, optimizing **X** is also a convex optimization problem. Thus, in this paper, we optimize **X** by fixing gene-pathway association matrix **B** ^(1)^ and drug-pathway association matrix **B** ^(2)^, and optimize gene-pathway association matrix **B** ^(1)^ and drug-pathway association matrix **B** ^(2)^ by fixing **X**.

#### Updating **X**

4 $$ {\displaystyle \begin{array}{l}\underset{\mathbf{X}}{\min }{\left\Vert \mathbf{Y}-\mathbf{XB}\right\Vert}_F^2\\ {}\mathrm{Subject}\  \mathrm{to}\ \sum \limits_i{\mathbf{X}}_{i,j}^2\le 1,\forall j=1,\dots, K,\end{array}} $$

where **Y** = [**Y** ^(1)^, **Y** ^(2)^] and **B** = [**B** ^(1)^, **B** ^(2)^]. We use gradient descent method [24] to solve the problem (4). We first calculate the derivative of matrix **X**, the detailed computation process can be written as follows:

5 $$ {\displaystyle \begin{array}{l}\frac{\partial {\left\Vert \mathbf{Y}-\mathbf{XB}\right\Vert}_F^2}{\partial \mathbf{X}}=-2\left(\mathbf{Y}-\mathbf{XB}\right){\mathbf{B}}^T\\ {}=2\left({\mathbf{XBB}}^T-{\mathbf{YB}}^T\right).\end{array}} $$

Thus, according to the update formula of gradient descent method, **X** can be updated by

6 $$ {\mathbf{X}}_{k+1}={\mathbf{X}}_k-2\mu \left({\mathbf{X}\mathbf{BB}}^T-{\mathbf{YB}}^T\right),k=0,1,2,\cdots, $$

where *μ* denotes a step size. And then at each iteration, we will project X_*k* + 1_ to the feasible region, that is, we will check if ${\sum}_i{\mathbf{X}}_{i,j}^2\le 1\left(\forall j=1,\cdots, K\right)$. If X_*k* + 1_ satisfies this condition, we will perform next step, if not, we will make it as X_*k* + 1_ = X_*k* + 1_/‖X_*k* + 1_‖. In addition, we also apply the Nesterov’s algorithm [25] to quicken the convergence speed of this algorithm.

#### Updating **B**^(1)^

In this paper, we assume that the relationship of genes and pathways is already known. Similar to [13], we also apply ordinary least squares (OLS) algorithm to solve gene-pathway association matrix **B** ^(1)^, that is, we decompose the original problem into *G* ^(1)^ separate OLS problems.

7 $$ {\displaystyle \begin{array}{l}\mathrm{For}\ q\in \left\{1,2,\cdots, {G}^{(1)}\right\},\\ {}\underset{{\mathbf{B}}_{:,q}^{(1)}}{\min }{\left\Vert {\mathbf{Y}}_{:,q}^{(1)}-{\mathbf{X}}_{:,{L}_{:,q}^{(1)}}{\mathbf{B}}_{L_{:,q}^{(1)},q}^{(1)}\right\Vert}_2^2.\end{array}} $$

According to the update formula of ordinary least squares algorithm, the gene-pathway association matrix **B** ^(1)^ can be updated as follows:

8 $$ {\displaystyle \begin{array}{r}{\mathbf{B}}^{(1)}\left({\mathbf{L}}^{(1)}\left(:,i\right),i\right)={\left[\mathbf{X}\left(:,{\mathbf{L}}^{(1)}\left(:,i\right)\right)\right]}^{-1}\left[{\mathbf{Y}}^{(1)}\left(:,i\right)\right]\ \\ {}i=1,2,\dots, {G}^{(1)},\end{array}} $$

where ${\mathbf{Y}}_{:,q}^{(1)}$ denotes the *q*-th column of gene-pathway association matrix **Y** ^(1)^, ${\mathbf{B}}_{L_{:,q}^{(1)},q}^{(1)}$ denotes a subvector of the *q*-th column vector of matrix **B** ^(1)^ corresponding to the non-zero elements of indicating matrix ${\mathbf{L}}_{:,q}^{(1)}$. ${\mathbf{X}}_{:,{L}_{:,q}^{(1)}}$ refers to a sub-matrix of matrix **X**, which consists of the columns corresponding to the non-zero elements of indicating matrix ${\mathbf{L}}_{:,q}^{(1)}$.

#### Updating **B**^(2)^

We observe each column of drug-pathway association matrix **B** ^(2)^, and decompose the optimization problem into *G* ^(2)^ separate *L*_2, 1_-norm minimization problems:

9 $$ {\displaystyle \begin{array}{l}\mathrm{For}\ q\in \left\{1,2,\cdots, {G}^{(2)}\right\},\\ {}\underset{{\mathbf{B}}_{:,q}^{(2)}}{\min }{\left\Vert {\mathbf{Y}}_{:,q}^{(2)}-{\mathbf{XB}}_{:,q}^{(2)}\right\Vert}_F^2+\lambda {\left\Vert {\mathbf{B}}_{:,q}^{(2)}\right\Vert}_{2,1}.\end{array}} $$

Note that the problem (9) cannot merge known drug-pathway association information. Thus, in order to use prior information, we modify the optimization problem (9) as follows,

10 $$ {\displaystyle \begin{array}{l}\mathrm{For}\ q\in \left\{1,2,\cdots, {G}^{(2)}\right\},\\ {}\underset{{\mathbf{B}}_{:,q}^{(2)}}{\min }{\left\Vert {\mathbf{Y}}_{:,q}^{(2)}-{\mathbf{XB}}_{:,q}^{(2)}\right\Vert}_F^2+\lambda \left({\left\Vert {\mathbf{B}}_{\left(1-{L}_{:,q}^{(2)}\right),q}^{(2)}\right\Vert}_{2,1}+{\left\Vert {\mathbf{B}}_{L_{:,q}^{(2)},q}^{(2)}\right\Vert}_2\right),\end{array}} $$

where similar to ${\mathbf{L}}_{i,j}^{(1)}$, ${\mathbf{L}}_{i,j}^{(2)}\in \left\{0,1\right\}$ is also an indicating matrix with the size of *K* × *G* ^(2)^. It is used to indicate drug-pathway matrix **B** ^(2)^. Besides, *λ* is a turning parameter to turn the sparsity of matrix **B** ^(2)^. Following, we will introduce the optimization process.

We first solve the part that the drug-pathway association **B** ^(2)^ is pointed by **L** ^(2)^, that is:

11 $$ \underset{{\mathbf{B}}^{(2)}}{\min }{\left\Vert {\mathbf{Y}}^{(2)}-{\mathbf{XB}}^{(2)}\right\Vert}_F^2+\lambda {\left\Vert {\mathbf{B}}^{(2)}\right\Vert}_2^2. $$

Note that we omit the notation in the objective function of problem (11). The objective function of problem (11) can be rewritten as the following equation.

12 $$ {\displaystyle \begin{array}{c}{\mathrm{J}}_1\left({\mathbf{B}}^{(2)}\right)={\left\Vert {\mathbf{Y}}^{(2)}-{\mathbf{X}\mathbf{B}}^{(2)}\right\Vert}_F^2+\lambda {\left\Vert {\mathbf{B}}^{(2)}\right\Vert}_2^2\\ {}\kern7.4em =\mathrm{Tr}\left[{\left({\mathbf{Y}}^{(2)}\right)}^T{\mathbf{Y}}^{(2)}\right]-2\mathrm{Tr}\left[{\left({\mathbf{Y}}^{(2)}\right)}^T{\mathbf{X}\mathbf{B}}^{(2)}\right]\\ {}\kern6.1em +\mathrm{Tr}\left[{\left({\mathbf{B}}^{(2)}\right)}^T\left({\mathbf{X}}^{\mathbf{T}}\mathbf{X}+\lambda \mathbf{I}\right){\mathbf{B}}^{(2)}\right],\end{array}} $$

where J_1_(⋅) is an auxiliary function and **I** ∈ R^*K* × *K*^ is a unit matrix. Then we compute the derivative of J_1_(**B** ^(2)^), and set its result to zero, we have

13 $$ {\displaystyle \begin{array}{c}\frac{\partial {\mathrm{J}}_1\left({\mathbf{B}}^{(2)}\right)}{\partial {\mathbf{B}}^{(2)}}=-2{\mathbf{X}}^T{\mathbf{Y}}^{(2)}+2\left({\mathbf{X}}^T\mathbf{X}+\lambda \mathbf{I}\right){\mathbf{B}}^{(2)}\\ {}=\mathbf{0}.\end{array}} $$

Thus, we can obtain:

14 $$ {\mathbf{B}}^{(2)}={\left({\mathbf{X}}^T\mathbf{X}+\lambda \mathbf{I}\right)}^{-1}{\mathbf{X}}^T{\mathbf{Y}}^{(2)}. $$

Then we solve the part that the drug-pathway association **B** ^(2)^ is pointed by 1 ‐ **L** ^(2)^. According to reference [26], we propose an efficient method to solve this problem. This problem can be described as follows:

15 $$ {\displaystyle \begin{array}{c}\underset{{\mathbf{B}}^{(2)}}{\min }{\left\Vert {\mathbf{Y}}^{(2)}-{\mathbf{XB}}^{(2)}\right\Vert}_F^2+\lambda {\left\Vert {\mathbf{B}}^{(2)}\right\Vert}_{2,1}\\ {}=\underset{{\mathbf{B}}^{(2)}}{\min }{\left\Vert {\mathbf{Y}}^{(2)}-{\mathbf{XD}}^{\hbox{-} 1/2}{\mathbf{D}}^{1/2}{\mathbf{B}}^{(2)}\right\Vert}_F^2\\ {}+\lambda \mathrm{Tr}\left[{\left({\mathbf{B}}^{(2)}\right)}^T{\mathbf{D}}^{1/2}{\mathbf{D}}^{1/2}{\mathbf{B}}^{(2)}\right],\end{array}} $$

where **D** is a diagonal matrix with the *i*-th diagonal element as:

16 $$ {d}_{ii}=\frac{1}{2{\left\Vert {\left({\mathbf{B}}^{(2)}\right)}^i\right\Vert}_2}. $$

Then we make **X**_1_ = **XD** ^‐1/2^ and ${\mathbf{B}}_1^{(2)}={\mathbf{D}}^{1/2}{\mathbf{B}}^{(2)}$. Thus, the problem (15) can be rewritten as follows:

17 $$ \underset{{\mathbf{B}}_1^{(2)}}{\min }{\left\Vert {\mathbf{Y}}^{(2)}-{\mathbf{X}}_1{\mathbf{B}}_1^{(2)}\right\Vert}_F^2+\lambda \mathrm{Tr}\left[{\left({\mathbf{B}}_1^{(2)}\right)}^T{\mathbf{B}}_1^{(2)}\right]. $$

The objective function of problem (17) can be rewritten as follows:

18 $$ {\displaystyle \begin{array}{c}{\mathrm{J}}_2\left({\mathbf{B}}_1^{(2)}\right)={\left\Vert {\mathbf{Y}}^{(2)}-{\mathbf{X}}_1{\mathbf{B}}_1^{(2)}\right\Vert}_F^2+\lambda \mathrm{Tr}\left[{\left({\mathbf{B}}_1^{(2)}\right)}^T{\mathbf{B}}_1^{(2)}\right]\\ {}\kern3.8em =\mathrm{Tr}\left[{\left({\mathbf{Y}}^{(2)}\right)}^T{\mathbf{Y}}^{(2)}\right]-2\mathrm{Tr}\left[{\left({\mathbf{Y}}^{(2)}\right)}^T{\mathbf{X}}_1{\mathbf{B}}_1^{(2)}\right]\\ {}+\mathrm{Tr}\left[{\left({\mathbf{B}}_1^{(2)}\right)}^T\left({{\mathbf{X}}_1}^T{\mathbf{X}}_1+\lambda \mathbf{I}\right){\mathbf{B}}_1^{(2)}\right].\end{array}} $$

Then we compute the derivative of ${\mathrm{J}}_2\left({\mathbf{B}}_1^{(2)}\right)$, and then set its result to zero, we obtain:

19 $$ {\displaystyle \begin{array}{c}\frac{\partial {\mathrm{J}}_2\left({\mathbf{B}}_1^{(2)}\right)}{\partial {\mathbf{B}}_1^{(2)}}=-2{\left({\mathbf{X}}_1\right)}^T{\mathbf{Y}}^{(2)}+2\left[{\left({\mathbf{X}}_1\right)}^T{\mathbf{X}}_1+\lambda \mathbf{I}\right]{\mathbf{B}}_1^{(2)}\\ {}=0.\end{array}} $$

Thus, we have:

20 $$ {\mathbf{B}}_1^{(2)}={\left[\left({{\mathbf{X}}_1}^T{\mathbf{X}}_1+\lambda \mathbf{I}\right)\right]}^{-1}{{\mathbf{X}}_1}^T{\mathbf{Y}}^{(2)}. $$

Therefore, we can obtain the updating formula of matrix **B** ^(2)^, that is, ${\mathbf{B}}^{(2)}={\mathbf{D}}^{-1/2}{\mathbf{B}}_1^{(2)}$. Note that diagonal matrix **D** depends on drug-pathway association matrix **B** ^(2)^. We summarize the alternating optimization algorithm for the *L*_2, 1_-iPaD method in Algorithm 1.
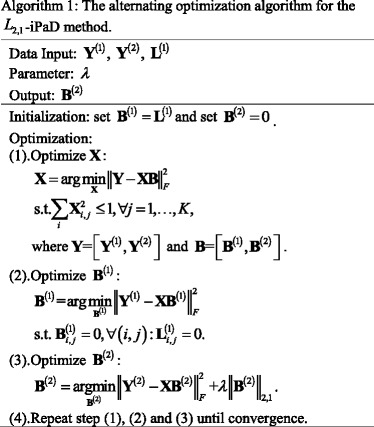


### Dealing with missing values

The gene expression data matrix **Y** ^(1)^ and drug related data matrix **Y** ^(2)^ in original data set have a few missing values. In order to strengthen the performance of our proposed method, we need to deal with missing values. Since each column of the gene-pathway association matrix **B** ^(1)^ and drug-pathway interaction matrix **B** ^(2)^ can be solved separately, the missing values in original data set can be removed in the process of updating matrix **B** ^(1)^ and **B** ^(2)^. However, we treat **X** as a whole matrix in updating matrix **X**. It is not easy to handle missing values, directly. Similar to [13], we use the soft-impute algorithm to handle the missing values during the process of updating **X**. The soft-impute algorithm can solve the incomplete matrix learning problem [27, 28]. Following, we will introduce the detailed process for handling missing values in the *L*_2, 1_-iPaD method.

Firstly, suppose that **Ω** ∈ {0, 1} is an indicating matrix with the size of *N* × (*G* ^(1)^ + *G* ^(2)^), and the matrix **Ω** can indicate observed values in the matrix **Y** (**Y** = [**Y** ^(1)^, **Y** ^(2)^]). And H_*Ω*_ is an operator, and when it projects the matrix **X** onto the space indicated by **Ω**, it satisfies the following formula:

21 $$ {\mathrm{H}}_{\varOmega }{\left(\mathbf{X}\right)}_{i,j}=\left\{\begin{array}{l}{\mathbf{X}}_{i,j},\mathrm{if}\ {\boldsymbol{\Omega}}_{i,j}=1.\\ {}0,\kern1.25em \mathrm{if}\ {\boldsymbol{\Omega}}_{i,j}=0.\end{array}\right. $$

Hence, the optimization problem for **X** can be expressed as follows:

22 $$ {\displaystyle \begin{array}{l}\underset{\mathbf{X}}{\min }{\left\Vert {\mathrm{H}}_{\varOmega}\left(\mathbf{Y}\right)-{\mathrm{H}}_{\varOmega}\left(\mathbf{XB}\right)\right\Vert}_F^2\\ {}\mathrm{s}.\mathrm{t}.\sum \limits_i{\mathbf{X}}_{i,j}^2\le 1,\forall j=1,\dots, K.\end{array}} $$

Then let **Ω**_1_ = 1 − **Ω**, which is used to indicate the missing values in the matrix **Y**, the problem (22) can be rewritten as:

23 $$ {\displaystyle \begin{array}{l}\underset{\mathbf{X}}{\min }{\left\Vert {\mathrm{H}}_{\varOmega}\left(\mathbf{Y}\right)-{\mathrm{H}}_{\varOmega}\left(\mathbf{XB}\right)\right\Vert}_F^2\\ {}=\underset{\mathbf{X}}{\min }{\left\Vert {\mathrm{H}}_{\varOmega}\left(\mathbf{Y}\right)-{\mathrm{H}}_{1-{\varOmega}_1}\left(\mathbf{XB}\right)\right\Vert}_F^2\\ {}=\underset{\mathbf{X}}{\min }{\left\Vert {\mathrm{H}}_{\varOmega}\left(\mathbf{Y}\right)-\left(\mathbf{XB}-{\mathrm{H}}_{\varOmega_1}\left(\mathbf{XB}\right)\right)\right\Vert}_F^2\\ {}=\underset{\mathbf{X}}{\min }{\left\Vert {\mathrm{H}}_{\varOmega}\left(\mathbf{Y}\right)+{\mathrm{H}}_{\varOmega_1}\left(\mathbf{XB}\right)-\mathbf{XB}\right\Vert}_F^2\\ {}\mathrm{s}.\mathrm{t}.\sum \limits_i{\mathbf{X}}_{i,j}^2\le 1,\forall j=1,\dots, K.\end{array}} $$

The detailed proving process can be found in [13, 27]. The problem (23) means that at every iteration, they will plug into ${\mathrm{H}}_{\varOmega_1}\left(\mathbf{XB}\right)$ for the next iteration. And this is exactly the main thought of the soft-impute method [27].

The specific step of the optimization algorithm is summarized in Algorithm 2.
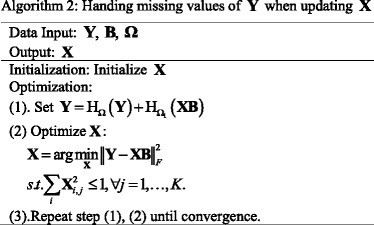


### Parameters selection and significance test

**Fig. 1:**
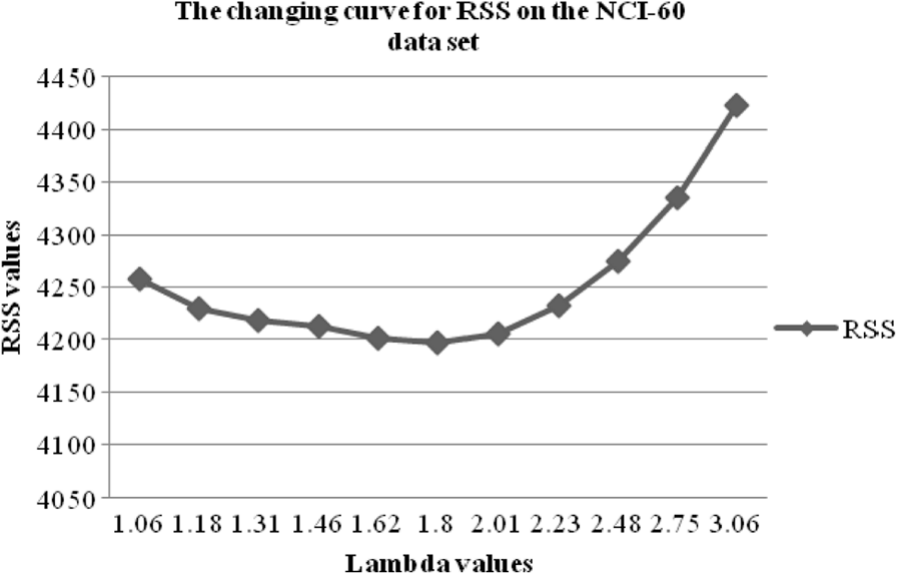
The changing curve for RSS on the NCI-60 data set

**Fig. 2:**
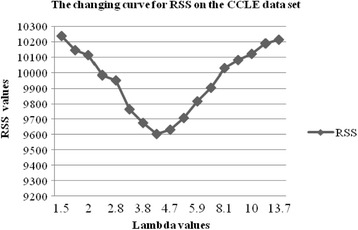
The changing curve for RSS on the CCLE data set

In the problem (3), *λ* is the only turning parameter, which is used to turn the sparsity of the drug-pathway interaction matrix **B** ^(2)^. The more important the drug-pathway associations is, the earlier the non-zero elements will become. Thus, we set the value of *λ* from producing the first non-zero elements in drug-pathway interaction matrix **B** ^(2)^ to 0.1. And then we use ten-fold cross-validation to obtain an appropriate *λ* value. Thus, we find an appropriate *λ* value according to the smallest residual sum of squares (RSS). The Figs. 1 and 2 show the changing curve for RSS on the NCI-60 and CCLE datasets, respectively. Thus, we can estimate the importance of the identified association pairs by recording the order of the values in the drug-pathway association matrix **B** ^(2)^ in which the values become non-zero. However, this identification method can not assess the significance of identified drug-pathway associations.

Therefore, we perform permutation test to assess the significance of identified drug-pathway associations after gaining an appropriate *λ*value, and calculate the *P* -values of every element in the drug-pathway association matrix **B** ^(2)^. Similar to [13], we also compute *P*-values by the following equation:

24 $$ {\mathrm{P}}_{i,j}=\frac{1}{\mathrm{T}}{\sum}_{t=1}^T\left(\left|{\mathbf{B}}_{i,j}^{(2)(t)}\right|\ge \left|{\mathbf{B}}_{i,j}^{(2)}\right|\right), $$

where ${\mathbf{B}}_{i,j}^{(2)(t)}$ denotes the value of drug-pathway association matrix **B** ^(2)^ in the *t*-th permutation. T denotes the overall number of permutations. And ${\mathbf{B}}_{i,j}^{(2)}$ is the estimated value in the original data.

## Results and discussion

In this section, we will show the experimental results on the real datasets, including the CCLE and NCI-60 datasets. And, in order to present the performance of our proposed method, we compare our proposed method with the iPaD algorithm.

### Results on the CCLE data set

In this subsection, in order to assess the performance of *L*_2, 1_-iPaD method, we apply this method to the CCLE data set in [13]. CCLE data set is downloaded from the CCLE project, which can provide public information as for the genomic data, analysis and visualization for about 1046 terms. The CCLE data set is made up of 480 cell lines (usually samples) with transcription data for 1802 genes and drug related data for 22 drugs covering 58 pathways. And those pathways are downloaded from KEGG database. And the drug related data are measured by area over the dose-response curve (“activity area”) since activity area can both express the potency and efficacy of chemical drugs. Besides, it has less unknown values [13]. In this paper, the known drug-pathway association pairs are regarded as validation information. And the prior information matrix **L** ^(2)^ is set to a zero matrix. In the iPaD method, the authors perform 2000 permutation to estimate *P*-values. The smaller the value of *P*-value is, the stronger the significance of identified drug-pathway association pairs becomes. And to be fair, we also perform 2000 permutation to assess the significance of identified drug-pathway association pairs in our method. Table 1 lists the P-values on CCLE data set for the *L*_2, 1_-iPaD and iPaD methods. Obviously, the *L*_2, 1_-iPaD method can mostly obtain smaller P-values than the iPaD method. In Table 1 the superior results are in italic type. Thus, our method is better than the iPaD method in identifying drug-pathway associations. Moreover, the *L*_2, 1_-iPaD method is a sparse optimization algorithm. Thus, nonzero elements in the drug-pathway association matrix **B** ^(2)^ are regarded as the drug-pathway association pairs. After applying our method to the CCLE data set, we discover that the *L*_2, 1_-iPaD method can identify 368 drug-pathway pairs, whose *p*-values are no more than 0.05, and 66 drug-pathway association pairs among them are verified in the CancerResource. But the iPaD method can only identify 88 drug-pathway association pairs, whose p-values are no more than 0.05, and 25 pairs among them are verified in the CancerResource. And then we compute the number of identified drug-pathway association pairs that P-values are no more than 0.005. For the iPaD method, it can identify 51 drug-pathway association pairs, and 16 drug-pathway association pairs among them can be verified in the CancerResource. And our proposed method can discover 53 association pairs with 16 drug-pathway associations verified in the CancerResource database. Tables 2 and 3 list the identification and verification rates of drug-pathway association pairs on the CCLE data set for the *L*_2, 1_-iPaD and iPaD methods. Note that in Table 2, we also compare our method with the iFad method. And the identification number denotes the number of drug-pathway association pairs, which posterior probabilities are no more than 0.9. The number of verification denotes the number of identified drug-pathway association pairs, which are validated in the CancerResource. The results of iFad is derived from the reference [13]. It is obvious that the performance of our method is better than the iFad method. In Table 1, we can discover that the drug of Nutlin-3 is associated with Chronic myeloid leukemia pathway. And, a study published in [29] said that the drug of Nutlin-3 is a tumor suppresser, which can up-regulate the expression of Notch1 in both lymphoid and myeloid leukemic cells. And we discover that PD-0332991 is a CDK 4/6 inhibitor [30] and can act on chronic myeloid leukemia [31]. In Table 1, LBW242 is associated with Chronic myeloid leukemia pathway, but their association is not validated in the CancerResource. A study published in 2007 says that LBW242 can effect on mutant FLT3-expressing cells in potentiating antileukemic therapies [32]. Therefore, our method can also identify drug-pathway association pairs which are not validated in CancerResource.

**Table 1:** The top 15 identified drug-pathway association pairs on CCLE data set to L_2,1_-iPaD and iPaD methods

Drug	KEGG pathway	*L*_2, 1_-iPaD	iPaD	Validated?
Sorafenib	Calcium signaling pathway	*0*	5.79E-04	Yes
Panobinostat	Pancreatic cancer	*0*	6.07E-04	No
LBW242	Chronic myeloid leukemia	*2.80E-44*	1.34E-10	No
Nutlin-3	Chronic myeloid leukemia	*1.74E-43*	4.82E-16	Yes
L-685458	Chronic myeloid leukemia	*4.33E-43*	3.20E-31	No
17-AAG	Chronic myeloid leukemia	*9.46E-43*	2.79E-20	No
AZD0530	Colorectal cancer	*1.62E-41*	3.05E-07	No
PD-0332991	Chronic myeloid leukemia	*6.93E-41*	1.38E-09	Yes
PHA-665752	Chronic myeloid leukemia	*1.09E-40*	1.97E-20	No
Paclitaxel	Chronic myeloid leukemia	*2.14E-38*	2.52E-16	No
AZD0530	Chronic myeloid leukemia	*7.12E-38*	5.12E-13	Yes
ZD-6474	Chronic myeloid leukemia	*1.62E-21*	1.23E-11	No
AZD0530	ErbB signaling pathway	*4.41E-16*	2.81E-05	Yes
RAF265	ECM-receptor interaction	1.26E-15	*0*	No
Erlotinib	Chronic myeloid leukemia	*5.69E-15*	1.98E-11	Yes

The superior results are in italic type

**Table 2:** The identification and verification rates on CCLE data set with the *P*-values < 0.05

Method	Number of identification	Number of verification	Verification rate	Identification rate
*L*_2, 1_-iPaD	368	66	*0.0517*	*0.2884*
iPaD	88	25	0.0196	0.0689
iFad^a^	39.4	4.8	0.0038	0.0309

Note: ^a^The results of iFad method are derived from the reference thirteen. And the identification number denotes the number of drug-pathway association pairs, which posterior probabilities are no more than 0.9. The number of verification denotes the number of identified drug-pathway association pairs, which are validated in the CancerResource

The superior results are in italic type

**Table 3:** The identification and verification rates on CCLE data set with the *P*-values < 0.005

Method	Number of identification	Number of verification	Verification rate	Identification rate
*L*_2, 1_-iPaD	53	16	*0.0125*	*0.0415*
iPaD	51	16	*0.0125*	0.0399

The superior results are in italic type

We note that combining different cancer types of data can increase the number of samples and can be better to identify common signals from different cancer. But, this operation may weaken the knowledge, which is specific to certain cancer types. Thus, in this paper, we apply the *L*_2, 1_-iPaD method to the lung cancer data set, which is extracted from the CCLE data set. And we also apply the iPaD method to analyze lung cancer data set. Table 4 lists the identification and validation rates on the lung cancer data set.

**Table 4:** The identification and verification rates on CCLE lung cancer data set with the *P*-values < 0.05

Method	Number of identification	Number of verification	Verification rate	Identification rate
*L*_2, 1_-iPaD	*95*	*12*	*0.0094*	*0.0745*
iPaD	57	8	0.0063	0.0447

The superior results are in italic type

### Results on the NCI-60 data set

We also apply our method on the NCI-60 data set, which has been used in [10, 13]. The NCI-60 data set is from the NCI-60 project, which consists of 60 human cancer cell lines with nine cancer types. The specific data pre-processed process can be found in [10]. The NCI-60 data set is made up of transcription and drug sensitivity data, which are all downloaded from the CellMiner database [33], and can be found from the URL of http://discover.nci.nih.gov/cellminer. This data set contains 57 cell lines from eight different cancer types and 1863 genes covering 58 KEGG pathways and 101 drugs. The drug sensitivity is measured by *GI*_50_ values, which is the minimum concentration of the drug needed to inhibit the growth of 50% [34]. As a consequence, the lower *GI*_50_values can manifest the drug-sensitive response, the higher *GI*_50_ values can manifest the drug-resistant response [5]. In the NCI-60 data set, we also use the ten-fold cross-validation to discover an appropriate *λ* value. And then we also perform 2000 permutations to obtain the *P*-values, which can estimate the significance of the drug-pathway associations. Table 5 lists the *P*-values on NCI-60 data set for the *L*_2, 1_-iPaD and iPaD methods. After applying our proposed method to the NCI-60 data set, we discover that the *L*_2, 1_-iPaD method can find 562 association pairs, with the *P*-values no more than 0.05, and 163 pairs among them are verified in the CancerResource. However, the iPaD method can only discover 247 drug-pathway association pairs with the P-values no more than 0.05, and among those drug-pathway associations only 74 drug-pathway pairs are verified in the CancerResource. And then we calculate the number of identified drug-pathway association pairs that P-values are no more than 0.005. The iPaD method can find 72 association pairs with 26 drug-pathway association pairs among them validated in the CancerResource database. But our proposed method can identify 89 drug-pathway association pairs with 33 association pairs among them validated in the CancerResource. Tables 6 and 7 list the identification and verification rates of drug-pathway association pairs on NCI-60 data for the *L*_2, 1_-iPaD and iPaD methods. From Tables 6 and 7, we can prove that our proposed method can discover more drug-pathway association pairs than the iPaD method. Note that in Table 6, we also compare our method with the iFad method. Similar to the results of CCLE data set, the identification number denotes the number of drug-pathway association pairs with posterior probabilities that are no more than 0.9. The number of verification denotes the number of identified drug-pathway association pairs, which are validated in the CancerResource. The results of iFad is derived from the reference [13]. In the NCI-60 data set, the performance of our method is superior than the iFad method. In our method results, Cell cycle pathway is related with Tiazofurin, which is a C-nucleoside, is converted in sensitive cells to the active metabolite, TAD, which tightly bound at the NADH site inhibited IMP DH activity [35]. The results of reference [36] may be utilized in cancer chemotherapy to combine Tiazofurin with biologic response modifiers which recruit quiescent leukemic cells into the cell cycle. And Selenazofurin is an IMPDH inhibitor. The reference [37] has introduced that Selenazofurin and Tiazofurin are due to a cell cycle block that causes the cells to accumulate in the S-phase. Lomustine is a kind of anti-cancer drugs. It is associated with Tight junction, which contributes to the barrier property of brain endothelial cells [38]. In Table 5, we can find that Mycophenolic Acid (MPA) is related with the Cell cycle pathway, but their association is not validated in the CancerResource. Similar to the CCLE data set, we also use published literatures to prove their associations. The authors in [39] demonstrate that in peripheral blood lymphocytes, MPA can lead to an inhibition for the cell cycle proliferation. As a consequence, in the NCI-60 data set, our method can also infer drug-pathway association pairs, which are not validated in the CancerResource.

**Table 5:** The top 20 identified drug-pathway association pairs on NCI-60 data set to L_2,1_-iPaD and iPaD methods

Drug	KEGG pathway	*L*_2, 1_-iPaD	iPaD	Validated?
Hydroxyurea	Neuroactive ligand-receptor interation	*0*	NAN	No
Rebeccamycin	T cell receptor signaling pathway	*4.12E-16*	4.65E-10	Yes
Tiazofurin	Cell cycle	*8.19E-11*	7.54E-07	Yes
Selenazofurin	Cell cycle	*1.75E-10*	2.78E-07	Yes
Mycophenolic Acid	Cell cycle	*2.61E-10*	2.52E-06	No
Lucanthone	Tight junction	*1.04E-08*	4.31E-06	Yes
Tanespimycin	Jak-STAT signaling pathway	*9.95E-07*	2.67E-04	No
Primaquine	Natural killer cell mediated cytotoxicity	*1.14E-06*	2.69E-04	No
Aminoglutethi-mide	Primary immunodeficiency	*1.30E-06*	1.16E-04	No
Geldanamycin	Gap junction	*7.89E-06*	1.87E-04	No
Diallyl Disulfide	Acute myeloid leukemia	*8.13E-06*	8.41E-05	No
Carmustine	Cell cycle	*8.68E-06*	4.58E-04	No
Lomustine	Tight junction	*1.06E-05*	2.64E-04	Yes
Bleomycin	Focal adhesion	*1.17E-05*	4.56E-04	No
Vitamin K 3	Metabolism of xenobiotics by cytochrome P450	*2.22E-05*	2.71E-04	No
Melphalan	T cell receptor signaling pathway	*2.64E-05*	6.16E-04	Yes
Tegafur	Gap junction	*6.73E-05*	5.60E-04	No
Chloroquine Phosphate	Tight junction	*7.12E-05*	8.76E-04	Yes
Aclacinomyci- ns	One carbon pool by folate	*1.03E-04*	5.41E-04	No
Tamoxifen	Pyrimidine metabolism	*1.12E-04*	1.92E-03	No

The superior results are in italic type

**Table 6:** The identification and verification rates on NCI-60 data set with the *P*-values < 0.05

Method	Number of identification	Number of verification	Verification rate	Identification rate
*L*_2, 1_-iPaD	562	163	*0.0278*	*0.0959*
iPaD	247	74	0.0126	0.0422
iFad^a^	123	25.2	0.0043	0.0210

Note: ^a^The results of iFad method are derived from the reference thirteen. And the identification number denotes the number of drug-pathway association pairs, which posterior probabilities are no more than 0.9. The number of verification denotes the number of identified drug-pathway association pairs, which are validated in the CancerResource

The superior results are in italic type

**Table 7:** The identification and verification rates on NCI-60 data set with the *P*-values < 0.005

Method	Number of identification	Number of verification	Verification rate	Identification rate
*L*_2, 1_-iPaD	89	33	*0.0056*	*0.0152*
iPaD	72	26	0.0044	0.0122

The superior results are in italic type

## Conclusions

Drug-pathway association identification is an important issue in pharmacology. In this paper, we develop an effective algorithm named “*L*_2, 1_-iPaD” to discover novel drug-pathway associations. In the optimization model, the objective function has only one turning parameter *λ*. Thus, our proposed method is nearly turning-free. To find the best performance of our method, we apply ten-fold cross-validation to discover an appropriate *λ* value. And to estimate the significance of the identified drug-pathway association pairs, we perform permutation test to calculate the *P*-values. For the purpose of assessing the performance of the *L*_2, 1_-iPaD method, we apply this method in the CCLE and NCI-60 datasets. The experimental results in the CCLE and NCI-60 datasets demonstrate that our proposed method can discover more drug-pathway association pairs than the iPaD method. And the *L*_2, 1_-iPaD method can identify more validated associations.

With the development of genomics and pharmacology, dealing with transcription and drug sensitivity data has become feasible. Our proposed method has tremendously improved the performance of the original algorithm. In the future, we are ready to propose more efficient and robust algorithms to handle the high-throughput drug related data. And the rapid growth of the high-throughput gene expression and drug related data is calling for more effective algorithms to solve the computational problems.
